# Supplementary material for: Comparative efficacy of different exercise interventions in patients with ankylosing spondylitis: a systematic review and network meta-analysis
Source: PeerJ. 2025 Nov 25;13:e20336. doi: 10.7717/peerj.20336 (PMC12662062; doi:10.7717/peerj.20336)
Supplement: Supplemental Information 1 [file peerj-13-20336-s001.docx]

# Appendix 1 Search strategy

Table 1 Search strategy in PubMed

| Step | Search strategy |
| --- | --- |
| #1 | ((("Spondylitis, Ankylosing"[Mesh])) OR (Ankylosing spondylitis [Title/Abstract])) OR (Ankylosing Spondylarthritis [Title/Abstract]) |
| #2 | Search (exercise [MeSH Terms]) OR ((((((((((exercises[Title/Abstract]) OR physical activit*[Title/Abstract]) OR training*[Title/Abstract]) OR danc*[Title/Abstract]) OR yoga[Title/Abstract]) OR taichi[Title/Abstract]) OR wuqinxi[Title/Abstract]) OR baduanjin[Title/Abstract]) OR yijinjing[Title/Abstract]) |
| #3 | Search ((randomized controlled trial [pt] OR controlled clinical trial [pt] OR randomized [tiab] OR placebo [tiab] OR  clinical trials as topic [mesh: noexp] OR randomly [tiab] OR trial [ti]) NOT (animals [mh] NOT humans [mh])) |
| #4 | #4 #1 AND #2 AND #3 |

# Appendix 2 The classifications of exercise interventions

To compare the effects of different types of exercise, we classified exercise interventions into the following broad categories: control group (CG, participants did not receive any structured exercise intervention and maintained their usual daily activities), aquatic aerobic exercise (AAE, aiming to improve cardiovascular fitness through water-based rhythmic activities such as water walking, water jogging, or water aerobics), aquatic stretching exercise (ASE, focusing on enhancing flexibility and joint mobility through a series of low-impact stretching movements performed in water), land aerobic exercise (LAE, traditional aerobic exercises performed on land, such as walking, jogging, or aerobic dance), land stretching exercise (LSE, flexibility-oriented movements performed on land to increase the range of motion and muscle elasticity), China health campaign (CHC, incorporating traditional Chinese wellness practices such as Tai Chi, Baduanjin, and Qigong to promote holistic health through controlled breathing, slow movements, and mental focus), and muscle exercise (ME, designed to improve muscular strength and endurance using bodyweight, resistance bands, or weights).

# Appendix 3 Transformation formulas for estimating the mean and standard deviation

(1) When calculating SD from M (confidence interval) for intervention or control group

a. the sample size in each group＞100

• SE= (upper limit-lower limit)/3.92

• SD=SE*N^ (1/2)

b. the sample size in each group≤100

• Input “tinv (1-0.95, N_1_-1)” in Microsoft Excel to obtain t

• SE= (upper limit-lower limit)/ t

• SD=SE*N^(1/2)

(2) When calculating SD from MD and P value between intervention or control group

a. reporting the exact p value

• Input “tinv (p, N_1_+N_2_-2)” in Microsoft Excel to obtain t

• SE=MD/t

• SD1=SE/N_1_^(1/2)

b. reporting only significant levels (e.g. P＜0.05 or P＞0.05)

• The conservative calculation is to take the upper bound P. (e.g. P＜0.05 is replaced 0.05)

(3) When calculating M (SD) from the m (interquartile range) for intervention or control group

a. the sample size＞25, M=m;

the sample size≤25, M=(𝑎+2m+𝑏)/4

b. the sample size≤15, SD=(((𝑎−2𝑚+𝑏)^2^/4+(𝑏−𝑎)^2^)/12) ^ (1/2)

the sample size 15<n≤70, SD=(𝑏−𝑎)/4

the sample size＞70, SD=(𝑏−𝑎)/6

(4) When calculating M (SD) from the m (interquartile range) for intervention or control group

a. M= (𝑞_1_ +𝑚+𝑞_3_)/3

b. calculation of SD

• Z=(0.25𝑁_1_+0.375)/(𝑁1+0.25)

• Input “norm. inv(1-z,0,1)” obtain μ

• SD=(𝑞_3_−𝑞_1_)/(2∗𝜇)

Note:

M= mean; SE=standard error of mean; SD=standard deviation; N=sample size; MD= mean difference; m=median; a= minimum value; b = maximum value; q1= first quartile; q3= third quartile

**Appendix 4**

**BASDAI**

**global, node, and loop inconsistencies**

**
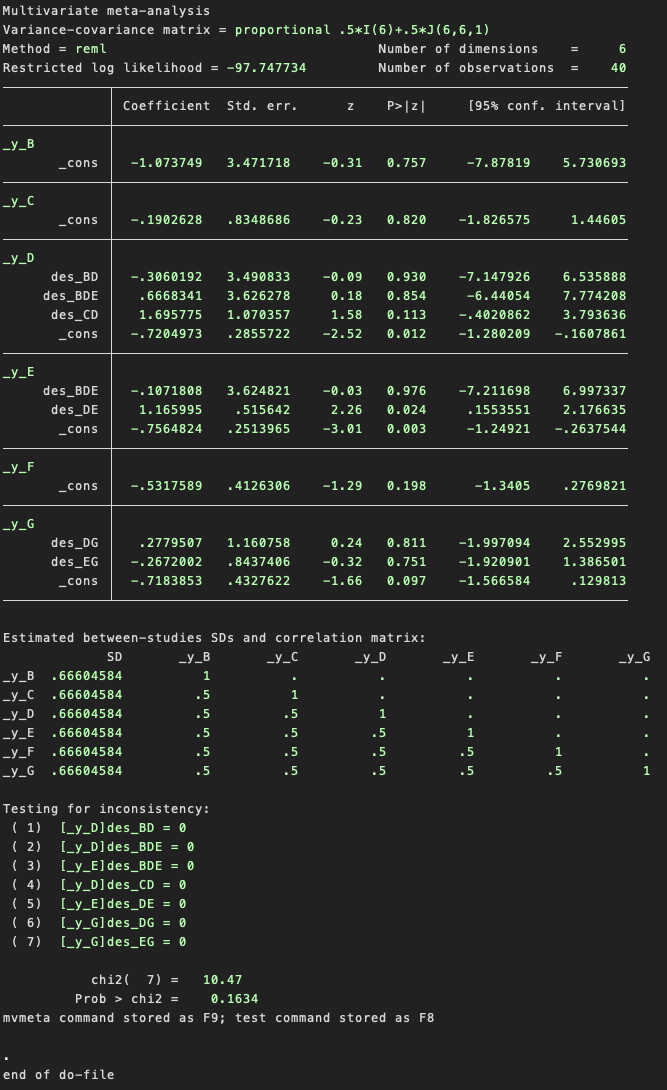
**

**
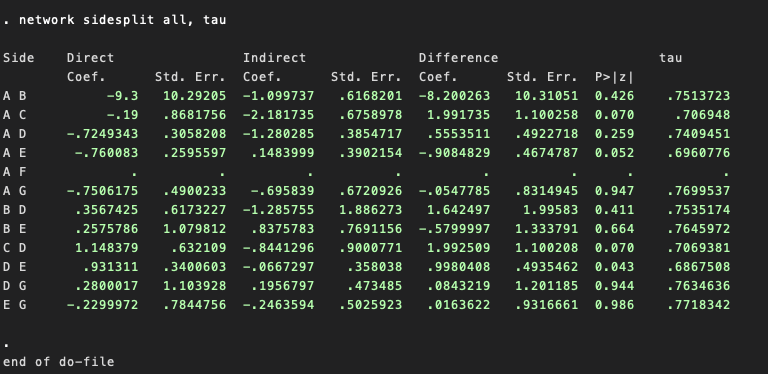
**

**
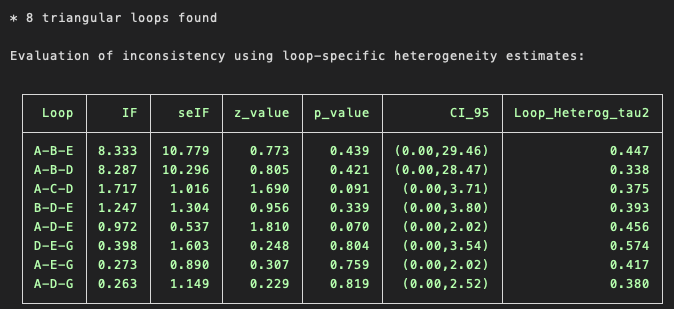
**

**BASFI**

**global, node, and loop inconsistencies**

**chi2(8) = 7.74**

**Prob > chi2 = 0.4595**

**
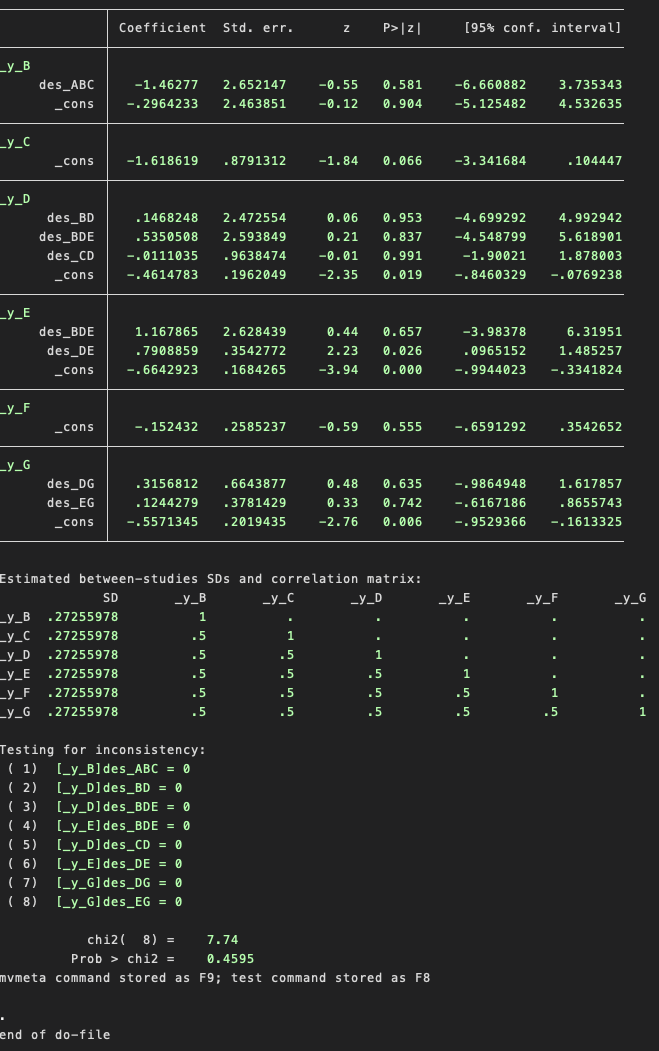
**

**
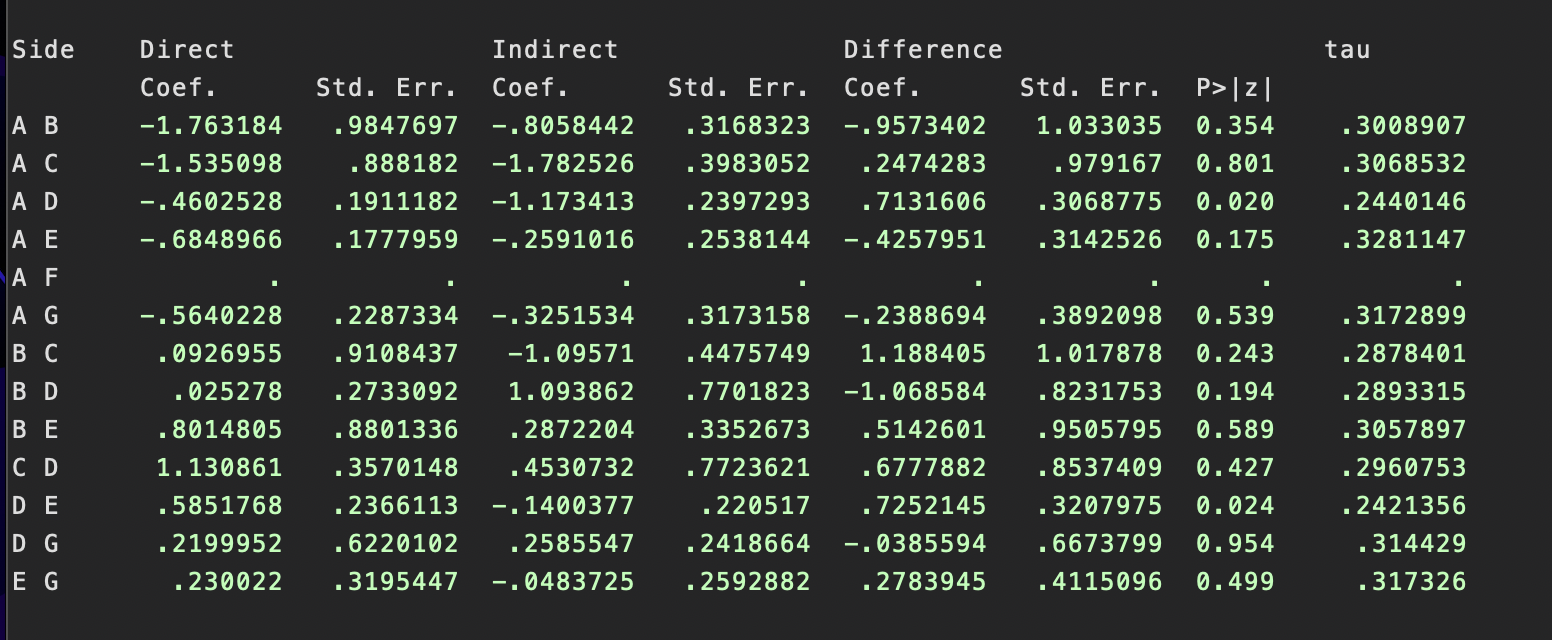
**

**
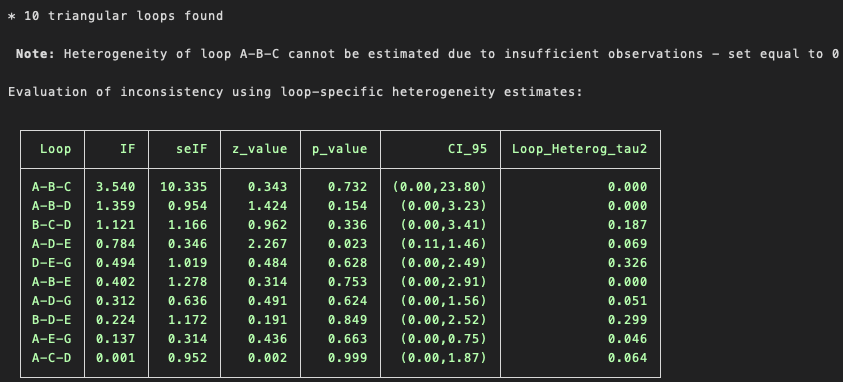
**

**BASMI**

**global, node, and loop inconsistencies**

**chi2(5) = 2.86**

**Prob > chi2 =0.7209**

**
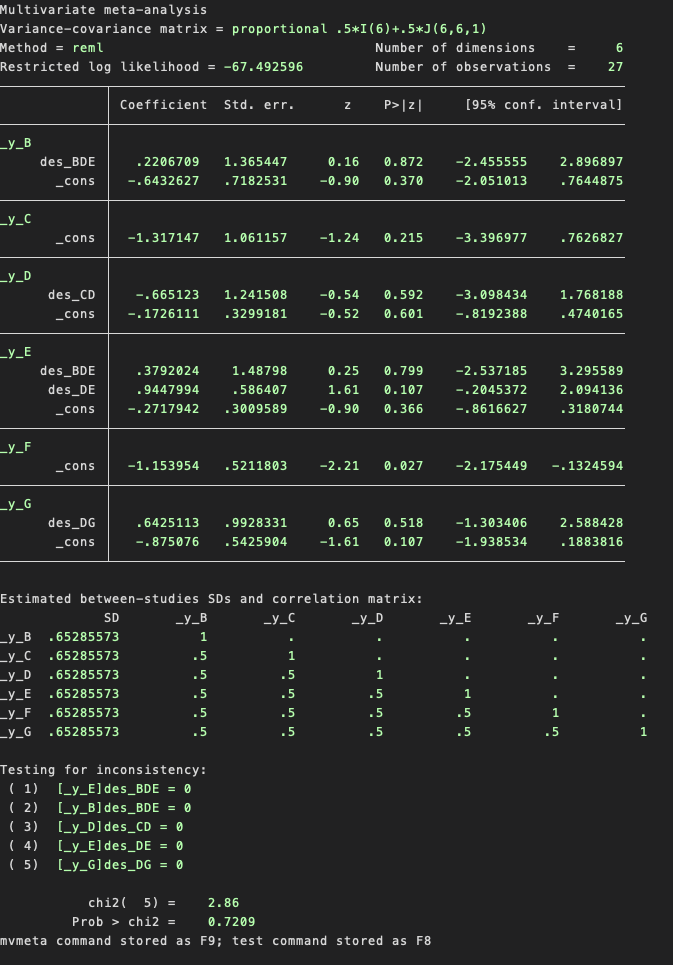
**

**
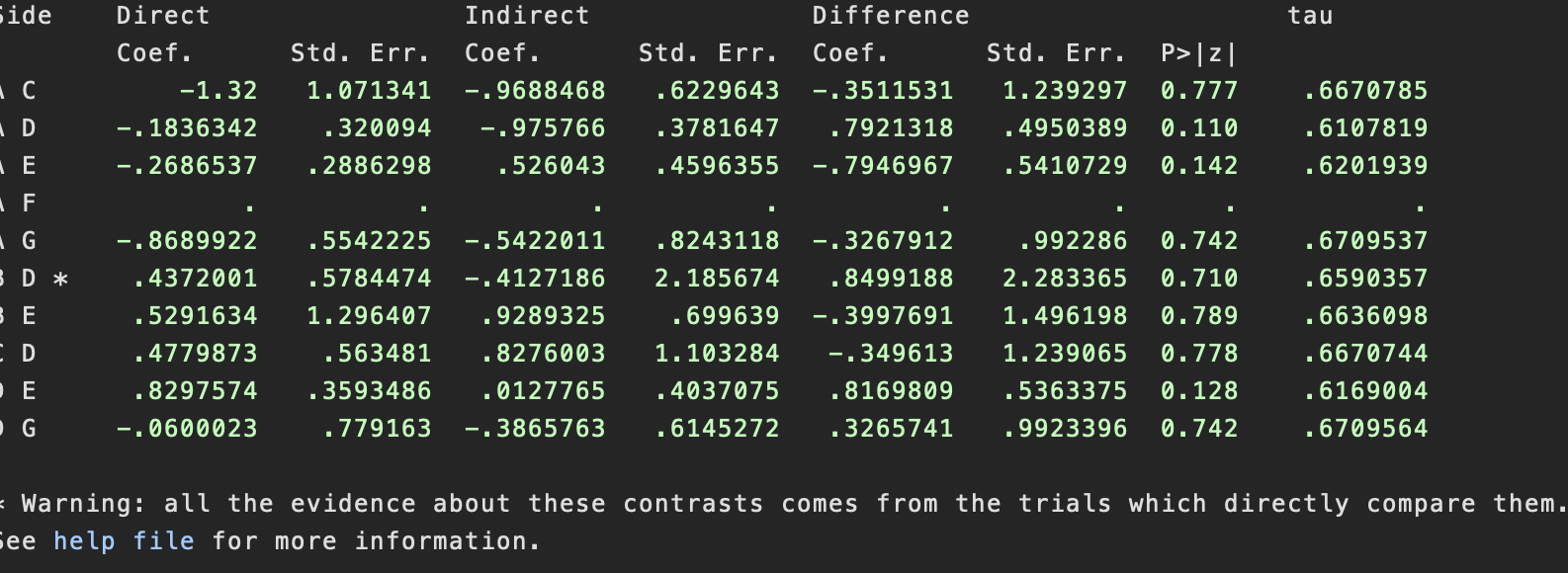
**

**
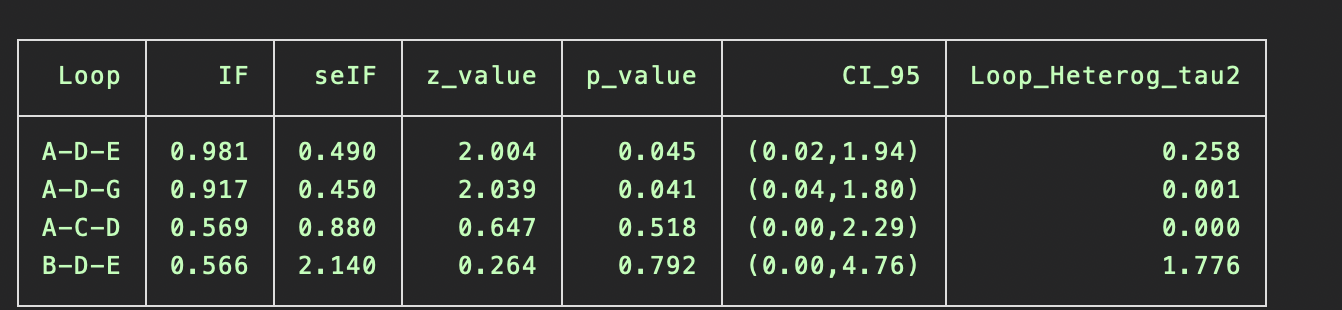
**

**ASQoL**

**global, node, and loop inconsistencies**

**chi2(1) =0.26**

**Prob > chi2 =0.6103**

**
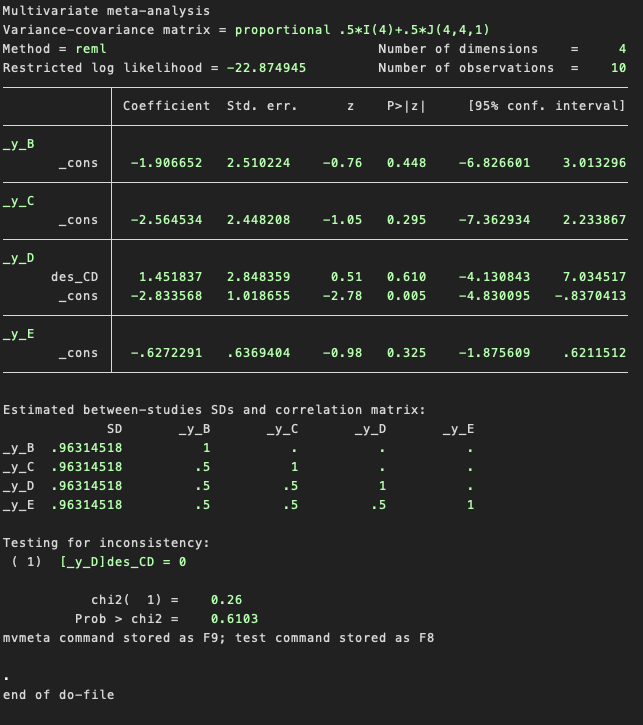
**

**
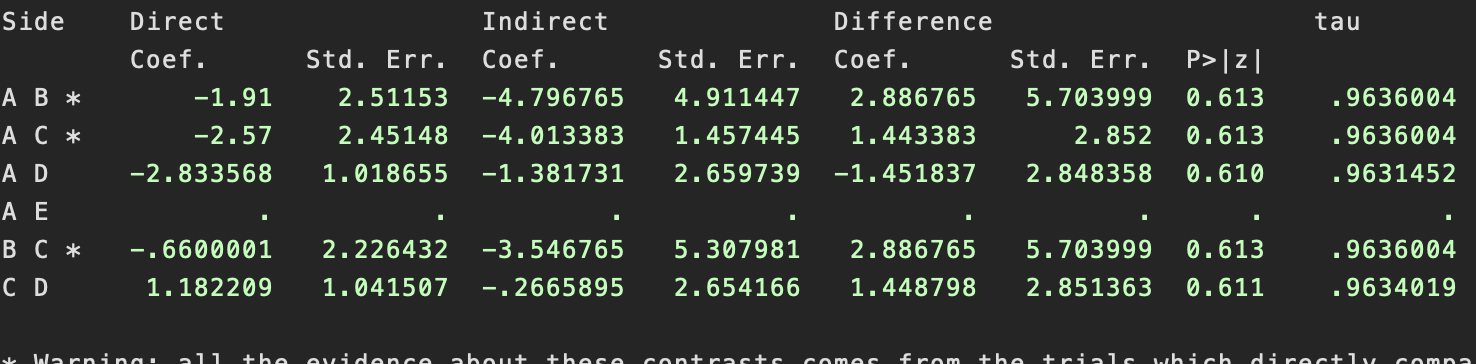
**

# Appendix 5

Fig. 6 (A) The funnel plot of BASDAI; (B) The funnel plot of BASFI; (C) The funnel plot of l BASMI; (D) The funnel plot of ASQoL

**
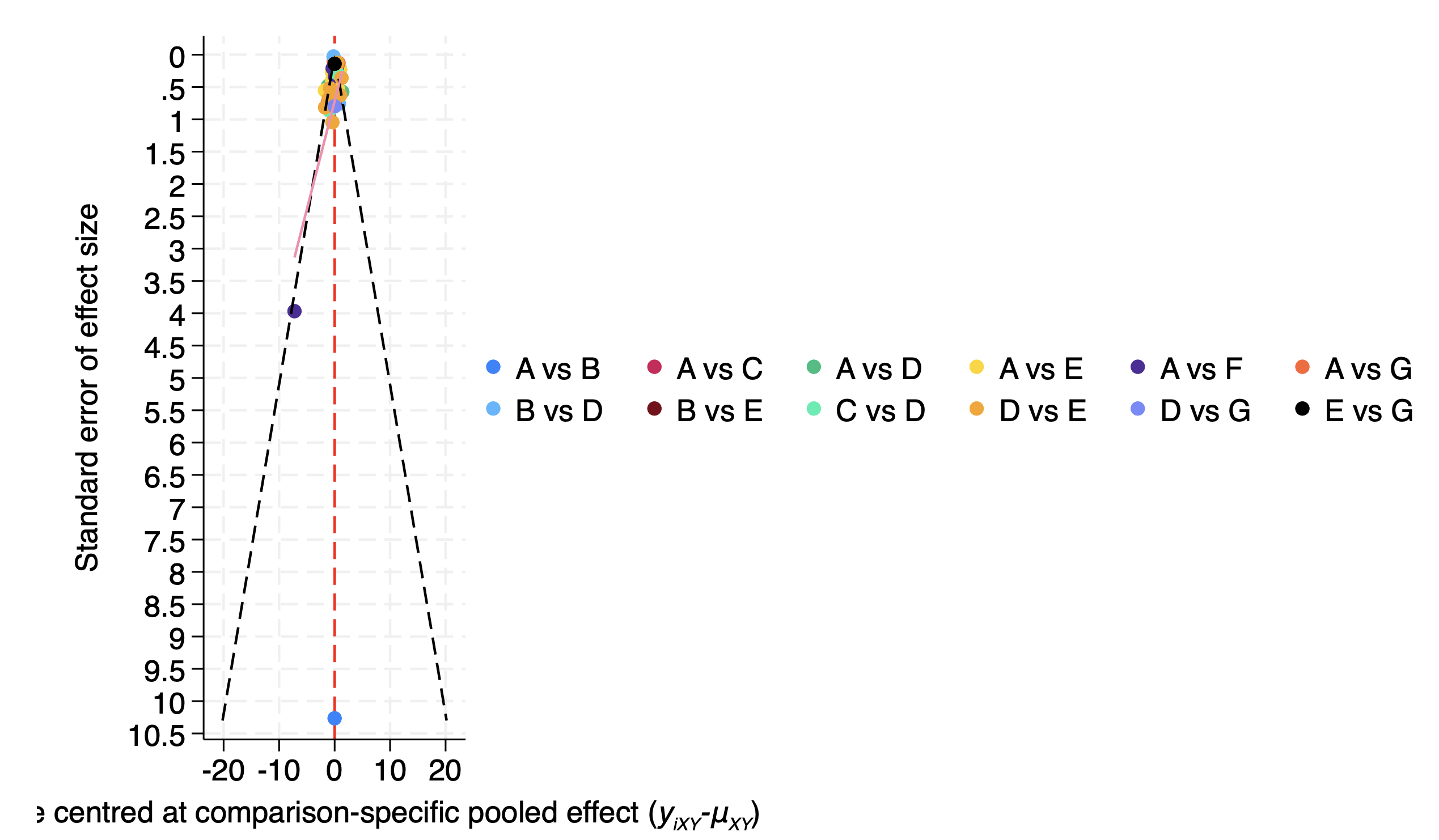
**

Figure 1(A) The funnel plot of BASDAI;


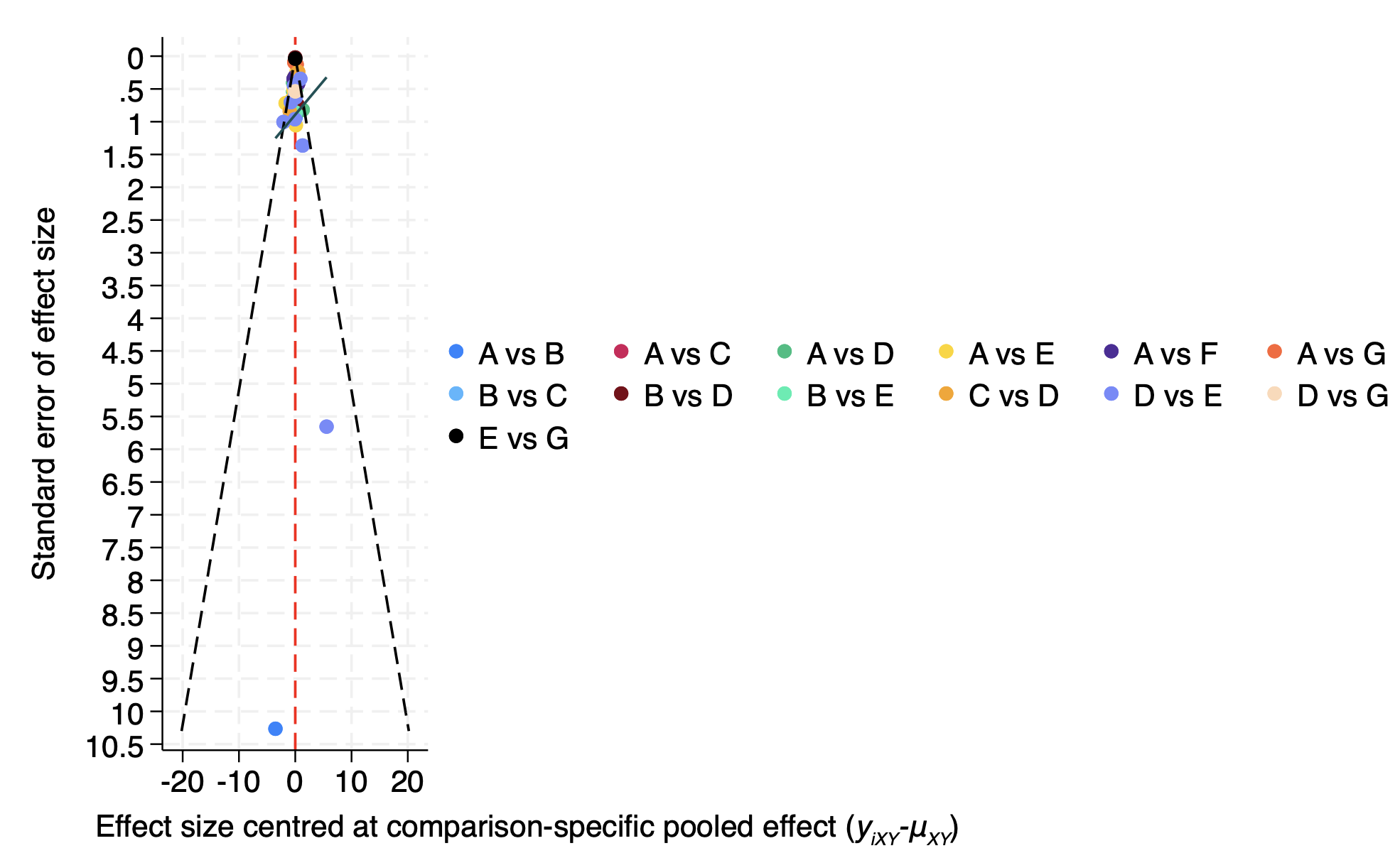


Figure 2 (B) The funnel plot of BASFI;


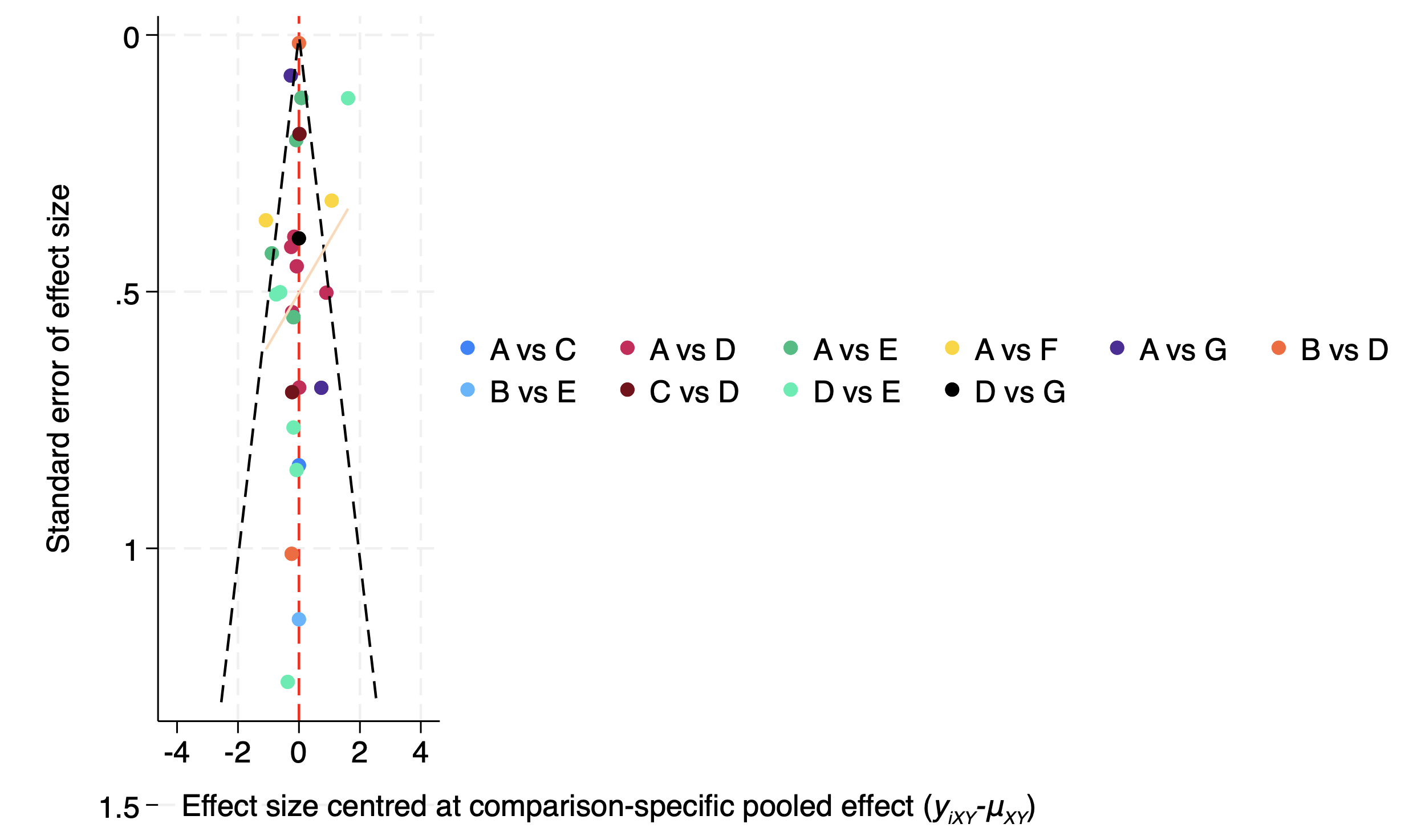


Figure 3(C) The funnel plot of l BASMI


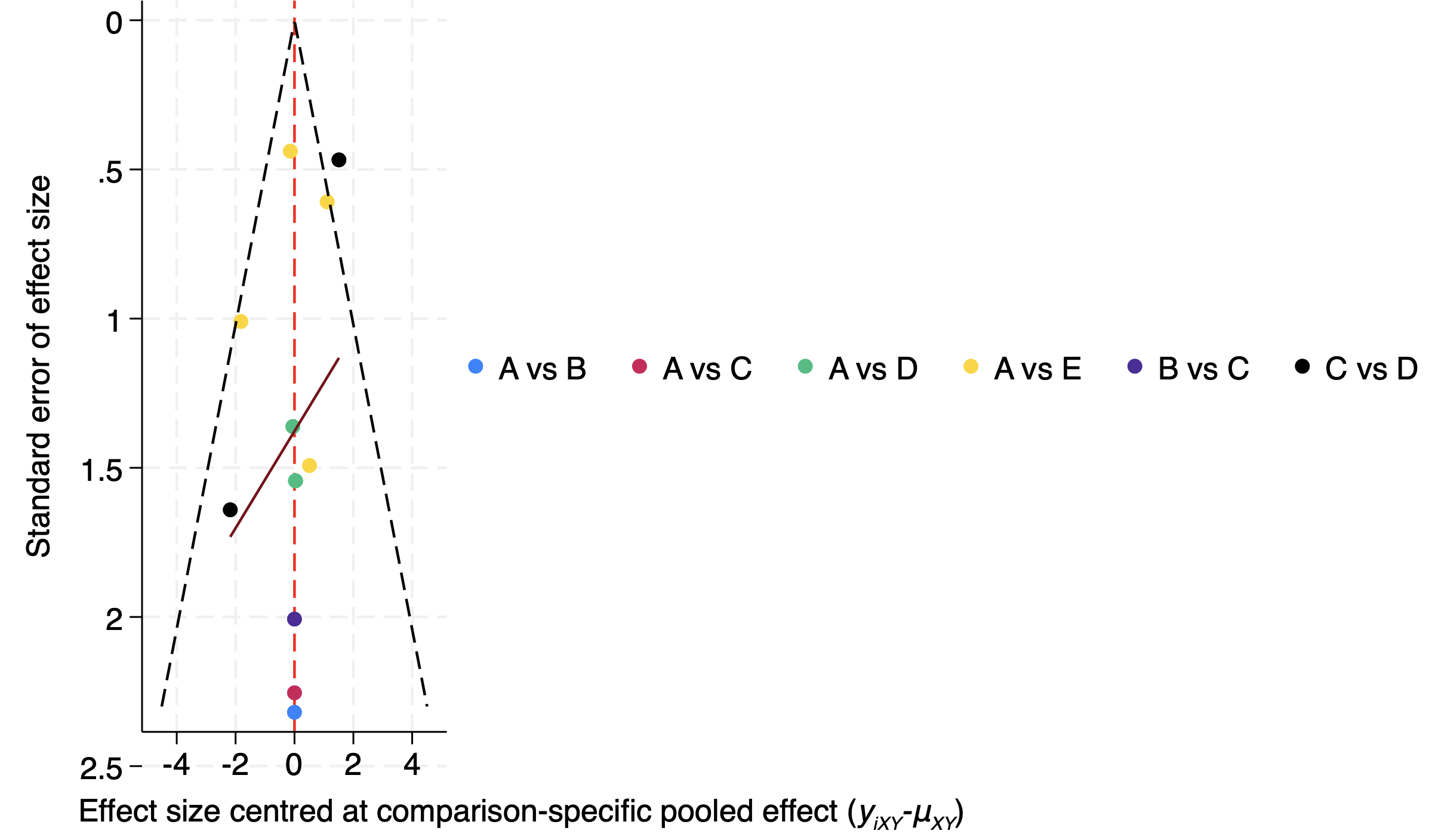


Figure 4 (D) The funnel plot of ASQoL
