# Supplementary material for: Comparative efficacy of different exercise interventions in patients with ankylosing spondylitis: a systematic review and network meta-analysis
Source: PeerJ. 2025 Nov 25;13:e20336. doi: 10.7717/peerj.20336 (PMC12662062; doi:10.7717/peerj.20336)
Supplement: Supplemental Information 3 [file peerj-13-20336-s003.docx]

Systematic Review and/or Meta-Analysis Rationale

1.For systematic reviews / meta-analyses, authors need to provide the following information:

Although exercise interventions are widely recognized as beneficial adjunctive treatments for ankylosing spondylitis (AS), the comparative efficacy of different exercise modalities remains unclear due to inconsistent findings across individual randomized controlled trials (RCTs). Therefore, we conducted a systematic review to comprehensively identify and synthesize the available evidence from RCTs. Given the diversity of interventions and the limited number of direct head-to-head comparisons, a network meta-analysis was employed to integrate both direct and indirect evidence, enabling the comparison of multiple interventions within a single analytical framework. This approach allowed us to rank the relative effectiveness of exercise types and provide evidence-based recommendations for clinical practice.

2.The rationale for conducting the systematic review / meta-analysis;

The contribution that it makes to knowledge in light of previously published related reports, including other meta-analyses and systematic reviews.

While previous systematic reviews and conventional meta-analyses have confirmed the general benefits of exercise in patients with ankylosing spondylitis (AS), they have been limited in scope, typically focusing on specific exercise types (e.g., aerobic vs. flexibility training), using pairwise comparisons only, or failing to evaluate the relative efficacy across a broader spectrum of exercise interventions. Moreover, these prior studies often lacked rigorous ranking methodologies and did not simultaneously incorporate all key clinical outcomes, such as BASDAI, BASFI, BASMI, and ASQoL.

Our study advances the field by being the first comprehensive network meta-analysis (NMA) to simultaneously compare multiple exercise modalities across four core outcome measures in AS. By leveraging direct and indirect evidence from 48 RCTs and applying a frequentist NMA framework, our study provides a hierarchical ranking of interventions using SUCRA values, offering new insights into which exercise interventions are most effective. For example, our findings highlight the consistent superiority of aquatic stretching exercise (ASE) across multiple domains, which was not established in previous literature.

Thus, our study fills a critical gap in the current evidence base and offers clinicians and policymakers an evidence-based reference for selecting optimal exercise prescriptions to manage AS.
