# Supplementary material for: Comparative efficacy of different exercise interventions in patients with ankylosing spondylitis: a systematic review and network meta-analysis
Source: PeerJ. 2025 Nov 25;13:e20336. doi: 10.7717/peerj.20336 (PMC12662062; doi:10.7717/peerj.20336)
Supplement: Supplemental Information 4 [file peerj-13-20336-s004.pdf]

Comparative efficacy of different exercise interventions in patients with ankylosing spondylitis: A systematic review and network meta-analysis

Lingkui Kong, Chuanwen Yu, Chaoxin Wang, Zhanpeng Meng

To enable PROSPERO to focus on COVID-19 submissions, this registration record has undergone basic automated checks for eligibility and is published exactly as submitted. PROSPERO has never provided peer review, and usual checking by the PROSPERO team does not endorse content. Therefore, automatically published records should be treated as any other PROSPERO registration. Further detail is provided here.

Citation
Lingkui Kong, Chuanwen Yu, Chaoxin Wang, Zhanpeng Meng. Comparative efficacy of different exercise interventions in patients with ankylosing spondylitis: A systematic review and network meta-analysis. PROSPERO 2025 CRD42025639115. Available from https://www.crd.york.ac.uk/PROSPERO/view/CRD42025639115.

PREVIEW

REVIEW TITLE AND BASIC DETAILS

Review title
Comparative efficacy of different exercise interventions in patients with ankylosing spondylitis: A systematic review and network meta-analysis

Review objectives
This meta-analysis of randomized controlled trials aims to evaluate the relative effectiveness of various exercise modalities on key outcomes, including disease activity, functional capacity, pain, and quality of life in patients with AS.

SEARCHING AND SCREENING

Searches
We will search articles in six electronic databases: PubMed, Web of Science, Embase, Cochrane Central Register of Controlled Trials, SPORTDiscus, and PsycINFO. We will search all English publications until January 15, 2025, without any restriction on countries or article types. The reference list of all selected articles will be independently screened to identify additional studies left out in the initial search.

Study design
Randomized controlled trials (RCTs) will be included. Randomized clinical trials will be included irrespective of blinding, publication status, or language.

ELIGIBILITY CRITERIA

Condition or domain being studied
Ankylosing spondylitis (AS) is a chronic inflammatory disease primarily affecting the spine and sacroiliac joints, often resulting in pain, stiffness, and reduced quality of life. Exercise is commonly recommended as a non-pharmacological intervention to manage symptoms and improve physical function in individuals with AS. However, current evidence regarding the effectiveness and comparative efficacy of different exercise interventions remains fragmented and inconsistent.

Population

Patients with ankylosing spondylitis (diagnosed by a clinician or using any recognized diagnostic criteria) will be included.

Intervention(s) or exposure(s)
Physical exercise was the main intervention (e.g., aerobic, resistance, and multicomponent exercise programs).

Comparator(s) or control(s)
Patients with ankylosing spondylitis who do not exercise

Context

OUTCOMES TO BE ANALYSED

Main outcomes
Key outcomes include disease activity, functional capacity, pain, and quality of life in patients with AS.

Additional outcomes
No Additional outcome(s).

Measures of effect

DATA COLLECTION PROCESS

Data extraction (selection and coding)
Two authors will independently extract data. Any disagreement will be resolved by discussion until a consensus is reached or by consulting a third author. The following data will be extracted: author, year of publication, country where the study was conducted, study period, original inclusion criteria, total number of people included in the survey, and exercise intervention time.

Risk of bias (quality) assessment
Two reviewers will independently assessesthe quality of the selected studies according to the Cochrane Collaboration's tool for the randomized controlled trials. Items will be evaluated in three categories: Low risk of bias, unclear bias and high risk of bias. The following characteristics will be evaluated: Random sequence generation (selection Bias) Allocation concealment (selection bias) Blinding of participants and personnel (performance bias) Incomplete outcome data (attrition bias) Selective reporting (reporting bias) Other biases Results from the sequestions will be graphed and assessed using Review Manager 5.3

PLANNED DATA SYNTHESIS

Strategy for data synthesis
Across all outcomes, a pairwise meta-analysis was first conducted to explore the effects of various exercise interventions compared with the control group. Effect sizes were estimated with SMDs and 95% confidence intervals (95%CIs) using postintervention scores by the random effects model.
Network meta-analyses for the primary outcomes were conducted using Stata software based on the frequentist framework. The network analysis pooled the individual study results. It combined both direct evidence from RCTs with head-to-head comparison and indirect evidence, where each of the two interventions was individually compared against a common comparator. Network diagrams were formed based on the network analysis, where each node represented an intervention, and the connecting lines between 2 nodes represented 1 or more RCTs in which the 2 interventions had been compared directly. The node size was weighted according to the number of participants who received the particular intervention, and the thickness of the lines connecting 2 nodes was weighted according to the number of studies that directly compared the interventions it connected. Random-effects models were applied, which accounted for heterogeneity caused by clinical and other factors across studies and provided a more conservative CI for pooled point estimates. SMDs and 95%CIs were calculated using the endpoint data after the intervention to

estimate the effect sizes of continuous outcomes. For clinical interpretation, effect sizes were classified as small (SMD < 0.40), moderate (SMD = 0.40–0.70), or large (SMD > 0.70), according to the Cochrane Handbook. The exercise interventions were ranked using the surface under the cumulative ranking curve (SUCRA) and mean rank.

Analysis of subgroups or subsets

We will consider subgroups such as sex and the severity of ankylosing spondylitis.

REVIEW AFFILIATION, FUNDING AND PEER REVIEW

Review team members

Mr Lingkui Kong. Heze University. China.

No conflict of interest declared.

Dr Chuanwen Yu (review guarantor and contact) Heze University. China.

No conflict of interest declared.

Professor Chaoxin Wang. Heze University. China.

No conflict of interest declared.

Dr Zhanpeng Meng. Shandong Normal University. China.

No conflict of interest declared.

Named contact

Dr Chuanwen Yu (yuchuanwen@hezeu.edu.cn). Heze University. China.

Review affiliation

Heze University

Funding source

No source of funds

TIMELINE OF THE REVIEW

Review timeline

Start date: 16 January 2025. End date: 31 March 2025

Date of first submission to PROSPERO

16 January 2025

Date of registration in PROSPERO

27 January 2025

CURRENT REVIEW STAGE

Publication of review results

The intention is not to publish the review once completed.

Stage of the review at this submission

Review stage	Started	Completed
Pilot work	✓	✓
Formal searching/study identification	✓	✓
Screening search results against inclusion criteria	✓	✓
Data extraction or receipt of IPD	✓	✓
Risk of bias/quality assessment	✓	✓
Data synthesis	✓	✓

Review status

The review is completed.

ADDITIONAL INFORMATION

PROSPERO version history

- Version 2.0, published 10 Aug 2025
- Version 1.1, published 27 Jan 2025
- Version 1.0, published 27 Jan 2025

Review conflict of interest

None known

Country

China

Revision note

We made these changes to improve areas that were previously incomplete, ensuring the content is more comprehensive and fully aligned with the review process and procedures.

Disclaimer

The content of this record displays the information provided by the review team. PROSPERO does not peer review registration records or endorse their content.

PROSPERO accepts and posts the information provided in good faith; responsibility for record content rests with the review team. The guarantor for this record has affirmed that the information provided is truthful and that they understand that deliberate provision of inaccurate information may be construed as scientific misconduct.

PROSPERO does not accept any liability for the content provided in this record or for its use. Readers use the information provided in this record at their own risk.

Any enquiries about the record should be referred to the named review contact
